# Sensing mechanism of a ratiometric near-infrared fluorescent chemosensor for cysteine hydropersulfide: Intramolecular charge transfer

**DOI:** 10.1038/s41598-020-57631-5

**Published:** 2020-01-20

**Authors:** Xiaofei Sun, Aihua Gao, Hongxing Zhang

**Affiliations:** 1 Laboratory of Theoretical and Computational Chemistry, Institute of Theoretical Chemistry, Jilin University, Changchun, 130023 China; 2 School of Physics and Optoelectronic Engineering, Ludong University, Yantai, 264025 China

**Keywords:** Excited states, Computational chemistry, Electronic structure of atoms and molecules

## Abstract

Previous studies have shown that the cysteine hydropersulfide (Cys-SSH) as the sulfur donor is crucial to sulfur-containing cofactors synthesis. Recently, a selective and sensitive near-infrared ratiometric fluorescent chemosensor Cy-DiSe has been designed and synthesized to detect Cys-SSH spontaneously. Herein, by means of the density functional theory (DFT) and time-dependent density functional theory (TD-DFT) approaches, the sensing mechanism has been thoroughly explored. According to our calculations, the experimental data have been reproduced. The results indicate the intramolecular charge transfer (ICT) is the reason for changes in fluorescence wavelengths. Compared with the chemosensor Cy-DiSe, the larger energy gap of Cy induced by ICT mechanism leads to the blue-shift of the absorption and emission spectra, which guarantees that Cy-DiSe can become a ratiometric fluorescent chemosensor to detect Cys-SSH.

## Introduction

As the widespread sulfur-containing biomolecules, reactive sulfur species (RSS) are essential in signal transduction and antioxidant physiological processes^1,2^. Among which, cysteine hydropersulfide (Cys-SSH), as the main source of hydropersulfide derivatives, is crucial to the synthesis of sulfur-containing cofactors, activating or inactivating enzyme activities, modulating cellular signaling, and regulating the cellular redox equilibrium^3–6^. Thus, it is important to obtain the concentration of intracellular Cys-SSH in real-time and *in situ*.

Compared with other biological detection technologies, fluorescent chemosensors have been exploited as the indispensable technique for detecting a variety of intracellular reactive species, due to their apparent advantages such as less invasiveness, operational simplicity, high sensitivity and selectivity^7–20^. Among these studies, ratiometric fluorescent chemosensors have attracted enormous attentions because they are able to function regardless of the external interferences, such as the concentration and polarity of solution, optic pollution and biological auto-fluorescence interference. Up to now, a mass of ratiometric fluorescent chemosensors have been synthesized by chemists^16,17,21–28^. It has been demonstrated that they can quantitatively detect bioactive molecules both in *vitro* and in *vivo*^17,24,25,28–30^. To the best of our knowledge, attentions are mainly focused on the novel synthesis of fluorescent chemosensors, and most of the sensing mechanisms are based on speculation^17,24,25,28–30^. Nevertheless, explorations on the sensing mechanisms of fluorescent chemosensors are also essential to design more effective fluorescent chemosensors. More importantly, with the help of computational chemistry, the relevant photophysical process and detailed sensing mechanism for fluorescent chemosensors can be comprehensively investigated.

Until now, numerous sensing mechanisms have been proposed and elucidated for designing fluorescent chemosensors, including the intramolecular charge transfer (ICT)^17,22,30–36^, the photoinduced electron transfer (PET)^8,9,37–40^, the excited state proton transfer (ESPT)^16,41–45^, and the fluorescence resonance energy transfer (FRET)^46–50^. Thereinto, ICT mechanism is usually adopted to design the ratiometric fluorescent chemosensors^17,21,22,29,34,51,52^. An ICT-based ratiometric fluorescent chemosensor can lead to the fluorescence spectrum displacement by enhancing or suppressing such an ICT process accompanying with partial charge transfer^7,32,53^.

**Figure 1:**
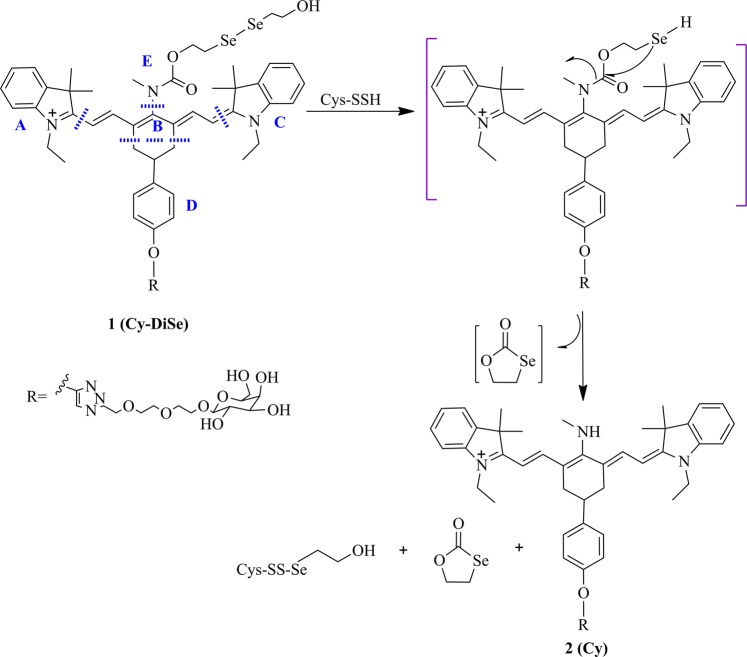
Proposed reaction mechanism for Cys-SSH detection.

Recently, Han *et al*.^17^. developed a near-infrared (NIR) ratiometric fluorescent chemosensor Cy-DiSe, which was designed according to the ICT mechanism, to detect Cys-SSH. Upon the detection of Cys-SSH, obvious changes in the spectra can be observed. The maximum value of absorption spectrum changes from 790 nm to 614 nm, while the maximum emission spectrum exhibits blue-shift from 797 nm to 749 nm. As shown in Fig. 1, after adding Cys-SSH to Cy-DiSe, a two-step reaction process occurs. Based on the reduction of diselenide, the intermediate is formed instantly, then through the intramolecular cyclization, the five-membered cyclic selenocarbonate is removed and the elimination product Cy is obtained ultimately. The authors only propose the reaction mechanism of modulating fluorescence changes, while the sensing mechanism still needs to be further clarified.

To elucidate the concrete sensing mechanism, we have carried out calculations based on the density functional theory (DFT) and time-dependent density functional theory (TD-DFT) approaches. We investigate the absorption and emission properties of Cy-DiSe and Cy involved in the sensing process. First, the ground (S_0_) and excited (S_1_) state geometries of the two molecules are optimized. Then, based on the optimized structures, the electronic transition energies, oscillator strengths, as well as the frontier molecular orbitals (FMOs) are analyzed. Besides, the D_CT_ and Δr indexes^54–56^, which can be adopted to measure the charge-transfer length and the hole-electron distance, are also provided to verify the ICT quantitatively. Our theoretical calculations not only reveal the ICT sensing mechanism, but also illustrate the reason why Cy-DiSe can serve as a ratiometric near-infrared fluorescent chemosensor for Cys-SSH.

## Results and Discussion

### Optimized geometries

**Figure 2:**
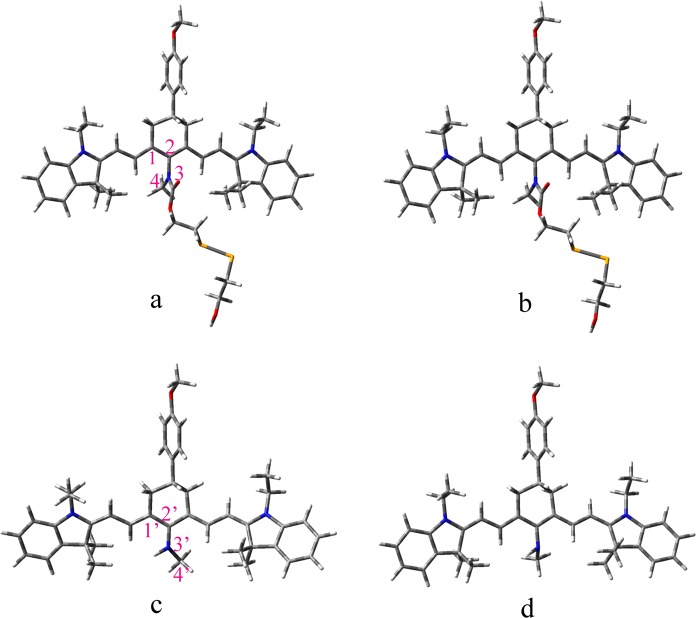
Optimized structures of Cy-DiSe and Cy in the ground (**a**,**c**) and excited (**b**,**d**) states, respectively.

The galactose group in Cy-DiSe enables it to target the liver, while it is speculated not to influence the relevant fluorescent properties. Therefore, the galactose group is replaced by methyl group for the sake of simplification. By adjusting the position of two indole rings in the cyanine skeleton, three optimized structures of chemosensor Cy-DiSe, namely, a, a1 and a2 (shown in Fig. S1), are obtained at the ground state. The energy of a is 0.10 eV and 0.19 eV lower than that of a1 and a2, and a is chosen as the most stable configuration for the following research. The optimized ground and excited state geometries of the Cy-DiSe and its elimination product Cy are displayed in Fig. 2. The atomic coordinates of these structures are provided in the electronic supplementary information (ESI). In the ground state, the calculated C_2_–N_3_ bond length and C_1_–C_2_–N_3_–C_4_ dihedral angle of Cy-DiSe are 1.44 Å and 90.9°, respectively. While in the excited state, the corresponding values are 1.44 Å and 89.8°, which are similar to the ground state. There is no obvious difference in geometries between the ground and excited states for Cy-DiSe. However, a noticeable change in the steric configuration of Cy has been observed. The bond length of C_2’_-N_3’_ is 1.36 Å in the ground state, which is lengthened to 1.41 Å in the excited state. Simultaneously, Cy exhibits significant conformational rotation with the dihedral angle of C_1’_-C_2’_-N_3’_-C_4’_ decreasing from 143.9° to 105.6° upon excitation.

### Absorption property analysis

Based on the optimized ground state structures, the low-lying singlet electronic transition energies and the corresponding oscillator strengths as well as the transition compositions of Cy-DiSe and Cy have been calculated and summarized in Table 1. In the experiment^17^, the initial absorption peak of Cy-DiSe lies in 790 nm. The addition of Cys-SSH results in a significant blue-shift, that is, with the increase of Cys-SSH concentration, the absorption peak at 790 nm gradually decreases in its height, while an increase of the new absorption peak at 614 nm is observed. The lowest electronic transition energies of Cy-DiSe and Cy have been calculated and shown in Table 1. Among all the calculated results, only the absorption spectrum of Cy-DiSe fails to be accurately reproduced because of the strong overestimation on the lowest excitation energy, regardless numerous methods have been tested (shown in Table S2). This result, though exhibiting difference comparing to experiment, is consistent with numbers of previous theoretical studies about cyanine^57–61^. The systematic and large overestimation of the lowest singlet excitation energy has been attributed to the difficulty of capturing the differential electron-correlation effects between ground and excited states, no matter what functionals, basis sets, or ground state geometries^57–61^ have been chosen. In fact, it is one of the theoretical signatures of cyanine that the obtained transition energy is rather insensitive to the selection of a specific (pure or hybrid) exchange-correlation functional^59,60^. It should also to note that, although the lowest singlet excitation energy is overestimated, the geometries and vibrational signatures in this study are accurate^60,61^. For Cy-DiSe, the first singlet-transition (S_0_ → S_1_) is the dominant transition with the largest oscillator strength of 2.2938, which is mainly assigned to the highest occupied molecular orbital (HOMO) → the highest occupied molecular orbital (LUMO). Similarly, the calculated results reveal that an intense S_0_ → S_1_ excitation with the largest oscillator strength of 2.0955 as the primary transition corresponds to HOMO → LUMO for Cy.

**Table 1:** Comparison of experimental and calculated absorption at the TD-DFT/B3LYP/6-311 G(d) level.

Compound	Electronic transition	Energy (nm/eV)	Experimental absorption (nm/eV)	*f*	Composition	CI
Cy-DiSe	S_0_ → S_1_	630/1.97	790/1.57	2.2938	HOMO → LUMO	98.2%
Cy	S_0_ → S_1_	566/2.19	614/2.02	2.0955	HOMO → LUMO	99.9%

**Figure 3:**
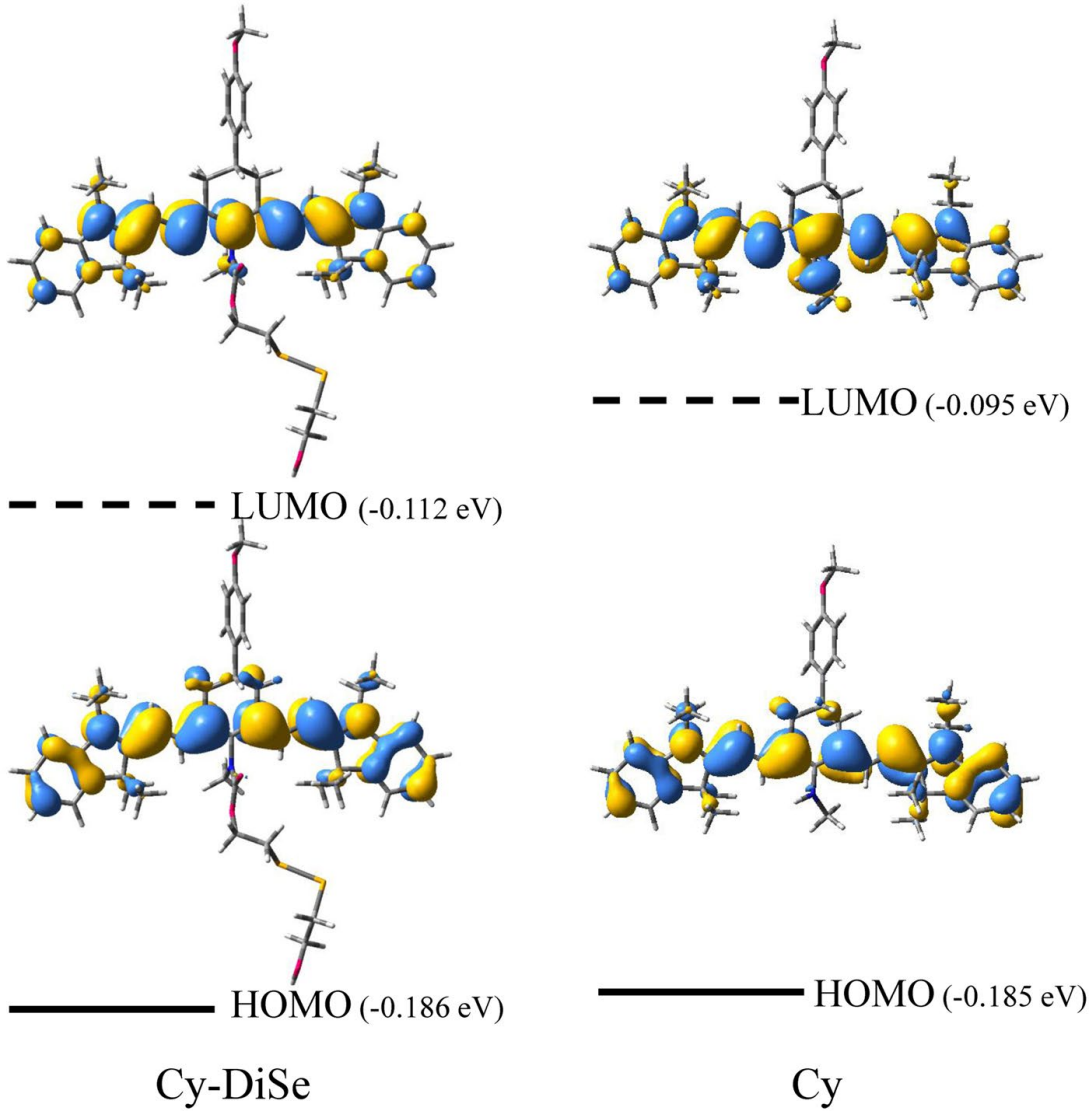
Calculated frontier molecular orbitals HOMO and LUMO in the absorption of Cy-DiSe and Cy, respectively.

Figure 3 displays the calculated frontier molecular orbitals for Cy-DiSe and Cy, so as to reveal the blue-shift of the emitted fluorescence after adding Cys-SSH. The MO distributions in HOMO for the two molecules are delocalized over the conjugated chain of cyanine, while the LUMO for both molecules share the similar distribution with that of HOMO. At the same time, the increase of the MO distribution on group E (see Fig. 1) accompanying with HOMO → LUMO transition implies that the S_1_ state of Cy own the ICT character. Figure 3 shows that the elimination of diselenide group leads to an increase of LUMO energy of Cy. Therefore, the larger energy gap of Cy induces the spectral blue-shift in contrast with Cy-DiSe. Such large obvious blue-shift between the spectra of Cy-DiSe and its elimination product Cy is essential for Cy-DiSe to act as a ratiometric fluorescent chemosensor for Cys-SSH.

### Molecular orbital compositions and indexes

In order to quantitatively describe the ICT character, atoms in both Cy-DiSe and Cy are categorized into five parts (A, B, C, D and E), respectively, among which the cyanine skeleton is subdivided into parts A, B and C (shown in Fig. 1). The contribution of each part to the FMOs is calculated based on the ground state and the corresponding results are listed in Table 2. Neither Cy-DiSe nor Cy exhibits charge transfer between HOMO and LUMO on parts B and C. However, compared with Cy-DiSe, an obvious charge transfer occurs on one of the indole ring A from cyanine skeleton of Cy. An electron transition is observed from 26.360% on HOMO to 19.083% on LUMO. Besides, for part D of Cy-DiSe and Cy, the MO distribution decreases about 3% from HOMO to LUMO. More importantly, part E of Cy exhibits obvious charge transfer compared to Cy-Dise with a MO distribution difference at around 9% between HOMO and LUMO, while in condition of Cy-DiSe, only about 2% is increased. From the results above, one can conclude that partial charge transfer from cyanine skeleton to methyl amino group exists in Cy upon excitation. Therefore, the S_1_ state of Cy shows the relatively distinct ICT character.

**Table 2:** Molecular orbital compositions in S_0_ state geometries.

Compound	MO	Composition (%)				
		A	B	C	D	E
Cy-DiSe	HOMO	22.436	49.340	22.170	5.425	0.629
	LUMO	23.433	49.555	23.620	0.804	2.588
Cy	HOMO	26.360	46.248	21.715	4.901	0.776
	LUMO	19.083	48.203	21.367	1.379	9.969

In addition, a quantitative analysis is performed to measure the hole-electron distance and the charge-transfer length by calculating the two indexes, namely Δr and D_CT_^56^. The calculated results of Δr and D_CT_ for Cy-DiSe and Cy are listed in Table 3, accompanying with the transferred charge (q_CT_) between the donor and acceptor groups. The values of Δr and D_CT_ are larger, the characteristic of charge separation is more apparent, that is, the ICT character is more significant. For Cy-DiSe, the small values of Δr and D_CT_ (about 0.5 Å) imply that there is almost no ICT character. Compared with Cy-DiSe, Cy exhibits the relatively obvious ICT character, which can be verified by the larger values close to 1 Å of Δr and D_CT_.

**Table 3:** Computed hole-electron distance, CT length and transferred charge for the S1 states of Cy-DiSe and Cy.

Compound	Δr (Å)	D_CT_ (Å)	q_CT_ (|e^−^|)
Cy-DiSe	0.45	0.57	0.40
Cy	0.80	0.89	0.48

### Emission property and sensing mechanism

**Figure 4:**
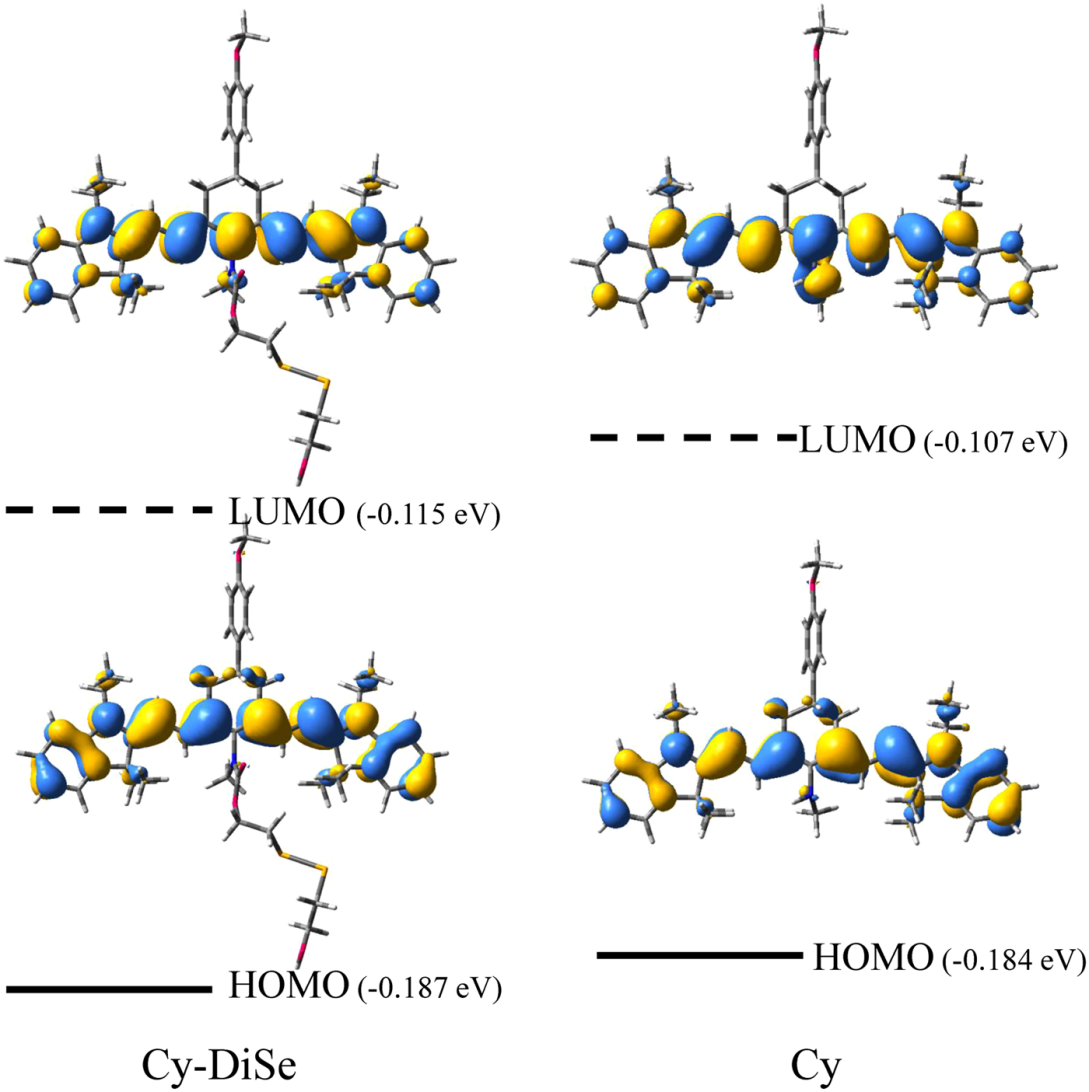
Calculated frontier molecular orbitals HOMO and LUMO in the emission of Cy-DiSe and Cy, respectively.

According to the viewpoint of analytical science, the fluorescent emission of a chemosensor is more sensitive for detection than the corresponding UV-Vis absorption^62^. Thus, the fluorescent characters of Cy-DiSe and its elimination product Cy are investigated to verify the potential of Cy-DiSe as a ratiometric fluorescent chemosensor for Cys-SSH. The sensing mechanism can thus be understood better. According to our calculations, all the involved excited states are the first singlet excited states. The optimized S_1_ state geometries of Cy-DiSe and Cy are displayed in Fig. 2, and the corresponding atomic coordinates are supplied in the ESI. Then, the emission properties of the two molecules are investigated using the TD-DFT method based on the optimized excited state structures. Table 4 shows their low-lying singlet electronic transition energies, oscillator strengths as well as the transition compositions and the relevant FMOs for the emission are given in Fig. 4.

**Table 4:** Comparison of experimental and calculated emission at the TD-DFT/B3LYP/6-311 G(d) level.

Compound	Electronic transition	Energy (nm/eV)	Experimental emission (nm/eV)	*f*	Composition	CI
Cy-DiSe	S_1_ → S_0_	772/1.61	797/1.56	2.6054	LUMO → HOMO	99.6%
Cy	S_1_ → S_0_	725/1.71	749/1.66	2.4889	LUMO → HOMO	99.4%

As shown in Fig. 4 and Table 4, the calculated emission wavelengths of Cy-DiSe and Cy are in agreement with the experimental measurements. For Cy-DiSe, the calculated emission wavelength is 772 nm and the experimental result lies in 797 nm. With the addition of Cys-SSH, the wavelength in experiment blue-shifts to 749 nm, while the calculated emission wavelength of Cy is 725 nm. Besides, for both experimental and calculated results, the emission spectrum of Cy all shows a blue-shift of about 50 nm compared with Cy-DiSe, which ensures that Cy-DiSe can act as the ratiometric fluorescent chemosensor. As can be seen from Table 4 and Fig. 4, the main relaxation transition for Cy-DiSe and Cy is S_1_ → S_0_ transition assigned to LUMO → HOMO with the largest oscillator strengths. Thus, the S_1_ state is a bright state which results in the strong fluorescence. Meanwhile, accompanying with LUMO → HOMO transition, the charge transfer between the nitrogen group and the cyanine skeleton implies that the S_1_ state of Cy exhibits the ICT character. The larger energy gap of Cy leads to the emission spectrum blue-shift compared with Cy-DiSe. Based on the calculated emission results, Cy-DiSe is confirmed again that it can serve as a ratiometric fluorescent chemosensor on detecting Cys-SSH.

According to the above calculated results, the sensing mechanism of the chemosensor Cy-DiSe can be depicted as follows. The added Cys-SSH triggers the O = C–N single bond cleavage reaction in Cy-DiSe and its elimination product Cy is obtained subsequently, which leads to the change of the electron density distribution. The locally excited state is responsible for the strong fluorescence emission, at the same time, the S_1_ state of Cy possesses ICT between the cyanine skeleton and nitrogen group. The larger energy gap of Cy results in the spectrum blue-shift compared with Cy-DiSe. Thus, Cy-Dise can serve as an excellent candidate of ratiometric fluorescent chemosensor when the ICT process is triggered by Cys-SSH.

## Conclusions

In summary, DFT and TDDFT methods have been applied to investigate the sensing mechanisms of the ratiometric fluorescent chemosensor Cy-DiSe and its elimination product Cy. The optimized geometries of the two molecules are obtained and there are no obvious differences between the ground and the excited states. It has been demonstrated that the theoretical calculated spectrum blue-shift, the strong fluorescence emission, and the large Stokes shift are all coincided with the experimental measurements. As the dominant transition for Cy-DiSe and Cy, S_0_ → S_1_ is assigned to HOMO → LUMO with the electron density mainly localized on the conjugated cyanine chain, which results in the strong fluorescence emission. Meanwhile, the partial intramolecular charge transfer from A and D groups of cyanine skeleton to the amino contained group E leads to the S_1_ state of Cy exhibit the ICT character. The calculated contribution results of each part to the molecular orbitals and the Δr and D_CT_ indexes provide further evidences that the S_1_ state of Cy possesses the ICT character. Because of the strong electron donating ability, amino can increase the LUMO energy of Cy, thus, the larger energy gap leads to the absorption spectrum blue-shift compared with Cy-DiSe. Accordingly, the calculated emission property results of Cy-DiSe and Cy are similar to those of absorption property. The large observable spectrum blue-shift supports that Cy-DiSe has the potential as a ratiometric fluorescent chemosensor when the ICT process is triggered by Cys-SSH. Our calculated results are helpful for the design of new ratiometric chemosensors with novel fluorescent properties based on the ICT sensing mechanism in future.

## Methods

In the present work, all theoretical calculations are carried out by using the Gaussian 09 program package^63^. The ground and first excited state geometries are optimized using the DFT/TD-DFT methods, respectively. Afterwards, the absorption and emission properties are further investigated by the TD-DFT methods. All geometric optimization calculations are completed without constrains for symmetry, bonds, angles or dihedral angles. The vibrational frequency analyses are carried out to ensure that each optimized structure is the real minimum without imaginary vibration frequency. In order to find the appropriate functional and basis set, a series of different functionals and basis sets have been tested shown in Table S2. Taking into account of the accuracy and computation efficiency, the Becke’s three-parameter hybrid exchange functional with Lee-Yang-Parr gradient-corrected correlation (B3LYP functional)^64–67^ and the 6-311 G(d)^68,69^ are selected for the following theoretical calculations. In all calculations, the solvent effect is included using the conductor-like polarizable continuum model (CPCM) with the dielectric constant of water (ε = 80.1) and the solute cavity is built with a special set of atomic radii as sug gested by Klamt^70,71^. Molecular orbital compositions and the indexes of Δr and D_CT_, which are used to indicate the hole-electron distance and the charge-transfer length, are calculated using the Multiwfn program^72^ to describe the ICT character quantitatively.

## Supplementary information

Supplementary information.
