# Supplementary material for: Sensing mechanism of a ratiometric near-infrared fluorescent chemosensor for cysteine hydropersulfide: Intramolecular charge transfer
Source: Sci Rep. 2020 Jan 20;10:711. doi: 10.1038/s41598-020-57631-5 (PMC6971067; doi:10.1038/s41598-020-57631-5)
Supplement: Supplementary file 1 — Supplementary information. [file 41598_2020_57631_MOESM1_ESM.pdf]

Supporting Information

for

Sensing mechanism of a ratiometric near-infrared fluorescent chemosensor for cysteine hydropersulfide: Intramolecular charge transfer

Xiaofei Sun[1], Aihua Gao[2], & Hongxing Zhang*[1]

[1]Laboratory of Theoretical and Computational Chemistry, Institute of Theoretical Chemistry, Jilin University, Changchun 130023, China. [2]School of Physics and Optoelectronic Engineering, Ludong University, Yantai 264025, China. Correspondence and requests for materials should be addressed to H.Z. (email: zhanghx@jlu.edu.cn)

Intersystem crossing (ISC)

As a heavy element, Se maybe facilitate the spin-orbital coupling (SOC) between a singlet state and a triplet state to promote the intersystem crossing. Therefore, it is necessary to consider the heavy-atom effect on the excited-state decay of Cy-DiSe. Based on the optimized ground-state geometry, the vertical excitation energies of the lowest ten singlet and triplet states are calculated, shown in Table S1. The results reveal that the T_1 state (0.94eV) lies below the S_1 state (1.97 eV) and the energy of T_2 (2.19 eV) is close to that of S_1. Subsequently, in order to explore whether the ISC can happen between the S_1 and T_1 or T_2, the SOC values are calculated based on the optimized lowest triplet excited-state geometry. It is worth mention that ISC between the singlet and triplet states can be conducted by direct SOC calculation. The results of $\langle T_1|H_{SOC}|S_0\rangle$, $\langle S_1|H_{SOC}|T_1\rangle$ and $\langle S_1|H_{SOC}|T_2\rangle$ are 0.23, 0.05 and 0.15 cm^{-1}, respectively. These small values of SOC demonstrate that the ISC process between these states is not likely to happen upon photo-excitation of Cy-DiSe.

No.	VVE (eV)	No.	VVE (eV)
S_1	1.97	T_1	0.94
S_2	2.66	T_2	2.19
S_3	2.97	T_3	2.61
S_4	3.02	T_4	2.65
S_5	3.12	T_5	2.90
S_6	3.14	T_6	2.97
S_7	3.33	T_7	3.10
S_8	3.55	T_8	3.12
S_9	3.56	T_9	3.14
S_{10}	3.64	T_{10}	3.24

Table S1. The vertical excitation energies (VVE) of the lowest ten singlet and triplet states for Cy-DiSe.

Compound	No.	Functional/Basis	Energy[a] (nm/eV)	*f*	Time
Cy-DiSe	1	B3LYP/TZVP	635/1.95	2.2496	3h55′
	2	BLYP/TZVP	673/1.84<br>640/1.94	1.4102<br>0.8134	2h3′
	3	BP86/TZVP	674/1.84<br>642/1.93	1.1939<br>1.0465	1h50′
	4	CAM-B3LYP/TZVP	635/1.95	2.2785	4h3′
	5	B3LYP-D3/TZVP	635/1.95	2.2496	3h42′
	6	PBE1PBE/TZVP	628/1.97	2.2831	4h16′
	7	B3P86/TZVP	634/1.96	2.2568	4h18′
	8	B3PW91/TZVP	633/1.96	2.2596	3h44′
	9	WB97XD/TZVP	639/1.94	2.2703	4h33′
	10	M062X/TZVP	624/1.99	2.2042	25h27′
	11	MPW1PW91/TZVP	628/1.97	2.2822	4h9′
	12	B3LYP/6-311G(d)	630/1.97	2.2938	1h2′
Cy	1	B3LYP/TZVP	571/2.17	2.0583	2h37′
	2	BLYP/TZVP	627/1.98<br>640/1.94	1.8300<br>0.8134	1h24′
	3	BP86/TZVP	626/1.98<br>642/1.93	1.8245<br>1.0465	1h17′
	4	CAM-B3LYP/TZVP	527/2.35	2.2171	3h1′
	5	B3LYP-D3/TZVP	571/2.17	2.0583	2h44′
	6	PBE1PBE/TZVP	558/2.22	2.1100	2h46′
	7	B3P86/TZVP	570/2.18	2.0631	2h34′
	8	B3PW91/TZVP	569/2.18	2.0642	2h34′
	9	WB97XD/TZVP	521/2.38	2.2320	2h53′
	10	M062X/TZVP	510/2.43	2.1866	1h6′
	11	MPW1PW91/TZVP	558/2.23	2.1104	2h43′
	12	B3LYP/6-311G(d)	566/2.19	2.0955	33′

a The values of experimental absorption are 790 nm and 614 nm for Cy-DiSe and Cy, respectively.

Table S2. The test results of TD-DFT calculation.

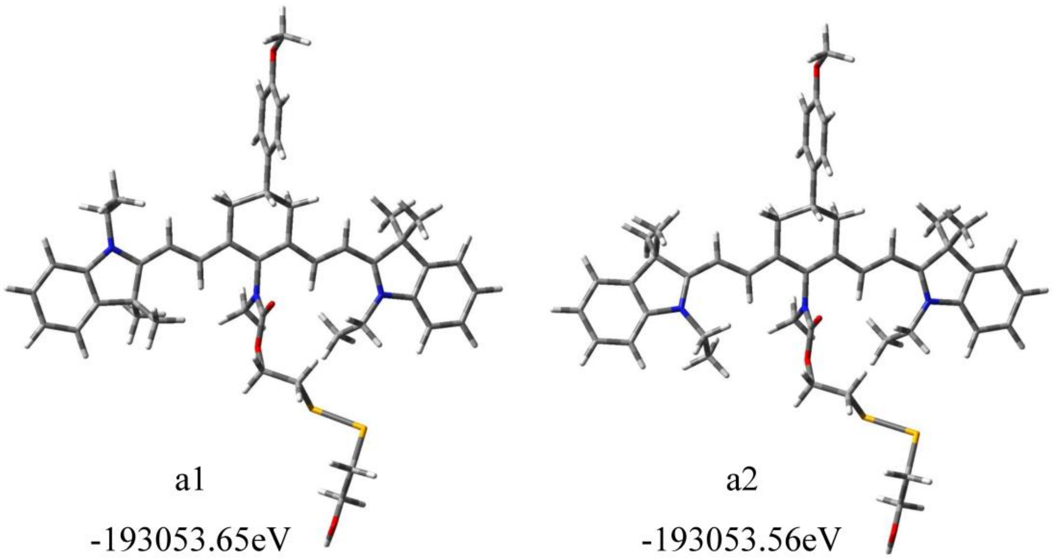

Figure S1. The calculated different configurations and the corresponding energy of Cy-DiSe in the S_0 state.

Appendix: List of coordinates

Coordinates for Cy-DiSe (S_0)

Coordinates for Cy (S_0)

Coordinates for Cy-DiSe (S_1)

Coordinates for Cy (S_1)

Coordinates for Cy-DiSe (T_1)

Coordinates for Cy-DiSe (S_0)

C	4.90852300	4.44203100	-1.02535600
C	5.24148100	3.08848700	-0.97562200
C	6.57023100	2.69856900	-1.03393100
C	7.55963600	3.68446400	-1.13770200
C	7.21072800	5.03570800	-1.18124100
C	5.87378000	5.44006100	-1.12498900
C	2.89713700	3.36465200	-0.84319200
H	6.84426700	1.64910000	-0.99944000
H	8.60428900	3.39601200	-1.18229300
H	7.98686500	5.78974400	-1.25809700
H	5.61671100	6.49194700	-1.15322300
C	3.97997100	1.44260200	0.44447400
H	4.83123800	0.75812900	0.43685500
H	4.07372500	2.09529500	1.31522000
H	3.07257400	0.84855000	0.55632500
C	3.97182800	2.26397800	-0.86516400
C	3.82747900	1.34727200	-2.10302000
H	3.80398000	1.93397600	-3.02402400
H	4.68330900	0.67037800	-2.15077300
H	2.92184500	0.74213600	-2.05662300

N	3.50525700	4.57311800	-0.96171800
C	2.83541400	5.88018200	-0.99354100
H	3.43819600	6.53252800	-1.62564700
H	1.87845700	5.75987200	-1.49986900
C	2.65427500	6.48928600	0.39663800
H	3.61665000	6.63524500	0.89194900
H	2.16693200	7.46298100	0.30594900
H	2.03198600	5.85391400	1.03040600
C	1.51465900	3.23521300	-0.71198100
H	0.94731600	4.15559900	-0.67310300
C	0.80478400	2.03439900	-0.62828800
H	1.35547500	1.10693200	-0.69832500
C	-0.58346000	1.91726700	-0.47671600
C	-1.19643500	0.64695800	-0.47527200
C	-1.46266600	3.14185200	-0.33293300
C	-2.58954900	0.45505100	-0.35849400
C	-2.72939800	2.84530700	0.48610500
H	-0.91090600	3.94885100	0.15361900
C	-3.48137700	1.65994500	-0.13764700
H	-3.92733200	1.96526400	-1.09287700
C	-3.61172800	4.06814500	0.65663300
C	-4.19326200	4.72259600	-0.44213000
C	-3.87291300	4.58774100	1.92540000
C	-4.99598200	5.84230000	-0.27451500
H	-4.01858600	4.35567100	-1.44890900
C	-4.67907900	5.71421400	2.11561700
H	-3.43898800	4.10676300	2.79759200
C	-5.24649800	6.34937900	1.00851200
H	-5.44088800	6.34212900	-1.12878900
H	-4.85146900	6.07840000	3.12057900

O	-6.04702500	7.45368600	1.06905600
C	-6.33255900	8.01598200	2.35122500
H	-6.97411300	8.87389100	2.16086500
H	-6.85910400	7.30036400	2.98904000
H	-5.41788800	8.34716700	2.85109600
N	-0.36183800	-0.51518800	-0.66504900
C	-0.05416800	-0.95923800	-2.03399900
H	1.02158600	-1.07294500	-2.16983200
H	-0.54592000	-1.90699800	-2.26236600
H	-0.41622300	-0.20017600	-2.72406700
C	0.03865100	-1.21261700	0.43442400
O	0.70502800	-2.34033100	0.07928600
O	-0.17487700	-0.88202700	1.58950100
C	1.19827900	-3.17909600	1.14955300
H	1.12823800	-4.19499900	0.76202300
H	0.54143300	-3.08014200	2.01382600
C	2.61782100	-2.82576600	1.54758500
H	2.69670900	-1.79294100	1.88267800
H	2.94411600	-3.49385300	2.34419700
Se	3.87450100	-3.08082800	0.01086300
Se	5.93559300	-2.70236100	1.09585100
C	6.35788800	-4.50480400	1.81390400
H	5.47231300	-4.88065000	2.32557100
H	7.13816500	-4.31805700	2.55599500
C	6.83866600	-5.47055900	0.74886800
H	6.06978500	-5.59674200	-0.02233100
H	7.74520500	-5.08284000	0.26932400
O	7.10337200	-6.71612000	1.40418400
H	7.39804900	-7.34791800	0.73721400
C	-3.14002600	-0.82878900	-0.47492900

H	-2.44277000	-1.63647200	-0.64411500
C	-4.50050300	-1.13953600	-0.40951900
H	-5.19381400	-0.32486400	-0.24710500
C	-6.70122100	-4.00411300	-0.57074000
C	-5.51806700	-4.72334900	-0.73791300
C	-5.55811000	-6.10202100	-0.87515200
C	-6.79986800	-6.74874400	-0.83787500
C	-7.97512400	-6.01527700	-0.66370100
C	-7.94642200	-4.62454900	-0.52552000
C	-5.05482400	-2.41412800	-0.52943300
C	-4.34273500	-3.76277300	-0.73720500
H	-4.64604300	-6.67476900	-1.00868800
H	-6.84843000	-7.82722600	-0.94277000
H	-8.93022100	-6.52873500	-0.63200100
H	-8.86462100	-4.06774500	-0.38338500
C	-3.61193600	-3.81733900	-2.09975400
H	-2.76498900	-3.13139100	-2.13204800
H	-3.23386200	-4.82879100	-2.26479400
H	-4.28971100	-3.56789200	-2.91909300
C	-3.38081900	-4.09846600	0.42582700
H	-2.51863500	-3.43145500	0.44533800
H	-3.89099400	-4.03177300	1.38919500
H	-3.01296800	-5.11977700	0.30429900
N	-6.39374000	-2.63104700	-0.46729800
C	-7.42774700	-1.60475500	-0.27828200
H	-8.32602500	-1.95969100	-0.78332500
H	-7.11410800	-0.70407900	-0.80467500
C	-7.71356600	-1.31396200	1.19491200
H	-6.82502800	-0.93628100	1.70515400
H	-8.49798900	-0.55712900	1.27029400

H	-8.05560900	-2.21175500	1.71442400
H	-1.74292500	3.51598800	-1.32609000
H	-4.31658600	1.39231400	0.51460000
H	-2.40271500	2.53494100	1.48547900

Coordinates for Cy (S_0)

C	7.12724500	-1.27797800	-0.09720200
C	6.65003900	-2.41750200	0.55268100
C	7.53735700	-3.31051300	1.13187100
C	8.91171600	-3.04630800	1.05816300
C	9.37444400	-1.90031200	0.40984700
C	8.48800800	-0.99317400	-0.17979700
C	4.84461800	-1.13449100	-0.29101300
H	7.17799800	-4.20161000	1.63700200
H	9.61839200	-3.73473100	1.50908600
H	10.44023600	-1.70239600	0.36155700
H	8.86077300	-0.10380900	-0.67354200
C	4.51994100	-2.43815100	1.88935500
H	4.85871500	-3.32180400	2.43538400
H	4.83148100	-1.55261600	2.44794800
H	3.42984000	-2.45924600	1.86050400
C	5.13298400	-2.44345100	0.47143300
C	4.67318100	-3.68413900	-0.32903700
H	5.09657300	-3.68036000	-1.33594800
H	5.01093100	-4.59006800	0.17985900
H	3.58775400	-3.73108000	-0.41318200
N	6.04049500	-0.54534700	-0.60668400
C	6.21005100	0.71136400	-1.34099100
H	7.15458000	0.64407000	-1.88199100

H	5.42753600	0.77262900	-2.09750500
C	6.19695900	1.94339400	-0.43511900
H	7.00179000	1.89766700	0.30216000
H	6.33897200	2.84370700	-1.03815600
H	5.24752000	2.03626700	0.09652800
C	3.62866400	-0.56628300	-0.62445900
H	3.66786700	0.39400000	-1.12180200
C	2.34830700	-1.10792200	-0.36772900
H	2.30881400	-2.09530800	0.07149700
C	1.13121100	-0.48154800	-0.57733000
C	-0.10726000	-1.13421000	-0.24434500
C	1.01328300	0.97552400	-0.96261300
C	-1.28300100	-0.34283100	0.00873200
C	0.26213900	1.71781500	0.16537900
H	1.99253400	1.42888000	-1.10860700
C	-1.16625200	1.15159300	0.26698300
H	-1.80277100	1.67195300	-0.45790600
C	0.27054900	3.22695700	0.00320600
C	-0.25698600	3.85449300	-1.13804700
C	0.81474100	4.04742200	0.99247100
C	-0.23812000	5.23503900	-1.27812500
H	-0.69067200	3.25789000	-1.93474700
C	0.84042400	5.44041600	0.87200900
H	1.23282200	3.59653900	1.88820700
C	0.31076600	6.04239500	-0.27147400
H	-0.64627200	5.71101200	-2.16384300
H	1.27210400	6.03290800	1.66879700
O	0.28040400	7.38815100	-0.50085200
C	0.82495200	8.25854300	0.49293300
H	0.69593900	9.26679100	0.10478300

H	0.28933500	8.16069600	1.44151500
H	1.88892100	8.06151900	0.65202800
N	-0.19613500	-2.48745400	-0.14464100
C	0.48230000	-3.48941700	-0.96532600
H	1.21244700	-4.06439100	-0.39072200
H	-0.26643300	-4.18172900	-1.35597700
H	0.98958000	-3.01308600	-1.80203200
C	-2.54136800	-0.92331700	-0.05292100
H	-2.59657300	-1.95424200	-0.37759800
C	-3.75871700	-0.26504300	0.21857900
H	-3.69854900	0.75566100	0.57376600
C	-7.31891600	-0.86792500	0.15992700
C	-6.94915200	-2.14639300	-0.26050900
C	-7.92033000	-3.10084200	-0.51763500
C	-9.26864600	-2.76063200	-0.34433500
C	-9.62316400	-1.48004600	0.08248000
C	-8.65153400	-0.50856000	0.34393500
C	-5.02752600	-0.80379700	0.08128000
C	-5.43464000	-2.22596300	-0.35306300
H	-7.64610100	-4.09809300	-0.84716500
H	-10.04009200	-3.49758700	-0.54033000
H	-10.66984500	-1.22833800	0.21808500
H	-8.94034600	0.47936700	0.68217000
C	-5.00450500	-2.52787400	-1.80721300
H	-3.91989200	-2.58365300	-1.90636600
H	-5.42095200	-3.48928800	-2.11689800
H	-5.37275000	-1.76043100	-2.49173000
C	-4.90280500	-3.30133200	0.62212600
H	-3.81607100	-3.38387300	0.58522800
H	-5.19525500	-3.07621200	1.65016800

H	-5.32252800	-4.27353000	0.35342400
N	-6.16175400	-0.08688200	0.33922600
C	-6.21172300	1.31177200	0.77528600
H	-7.13395100	1.73950400	0.38117200
H	-5.39407400	1.84787600	0.29356900
C	-6.15222600	1.46761500	2.29516100
H	-5.22277700	1.06169600	2.70022500
H	-6.20380500	2.52732300	2.55711900
H	-6.98996200	0.95739100	2.77597400
H	0.46754000	1.09021800	-1.90659500
H	-1.58066800	1.39824400	1.25180900
H	0.79104900	1.48892600	1.09687900
H	-1.00521700	-2.83900100	0.34437800

Coordinates for Cy-DiSe (S_1)

C	4.99285100	4.39178300	-1.06824100
C	5.33826000	3.03327000	-1.03472200
C	6.66697100	2.65333200	-1.11878000
C	7.64963100	3.64578300	-1.23317100
C	7.29242600	4.99817700	-1.26134600
C	5.95868100	5.39666900	-1.17863700
C	2.99924200	3.28852600	-0.86037600
H	6.94806400	1.60538000	-1.09664500
H	8.69491400	3.36385600	-1.29852700
H	8.06482400	5.75516600	-1.34678800
H	5.69741400	6.44769700	-1.19409700
C	4.11403800	1.36006300	0.39015300
H	4.96366700	0.67396600	0.35659100
H	4.22605800	2.00192500	1.26669400

H	3.20738600	0.76667100	0.51133800
C	4.07769300	2.19904000	-0.90998200
C	3.92006100	1.28616700	-2.15146000
H	3.88581000	1.87700200	-3.06941800
H	4.77413700	0.60768200	-2.21213800
H	3.01323700	0.68338500	-2.09515600
N	3.60624400	4.51546800	-0.97864400
C	2.92679300	5.81621800	-0.98190100
H	3.51561900	6.48528100	-1.60972100
H	1.96395900	5.69661600	-1.47768000
C	2.75681100	6.40405300	0.42030900
H	3.72426000	6.55581500	0.90371200
H	2.25575000	7.37242000	0.34809500
H	2.15127900	5.75201000	1.05323400
C	1.62096100	3.15354000	-0.70958400
H	1.05660700	4.07603400	-0.66050200
C	0.89628900	1.95487800	-0.62214600
H	1.43033100	1.01880000	-0.70539700
C	-0.49875900	1.86575500	-0.45827800
C	-1.15246800	0.60855500	-0.46038800
C	-1.34453100	3.10887800	-0.30313000
C	-2.55744600	0.45586900	-0.35403400
C	-2.62388200	2.84063100	0.50502500
H	-0.77427500	3.90033400	0.18652000
C	-3.40531000	1.69086300	-0.14775400
H	-3.80188400	2.01949500	-1.11758300
C	-3.46724700	4.08794200	0.69082900
C	-4.02575500	4.77712300	-0.39869600
C	-3.71286300	4.59651700	1.96727900
C	-4.79106600	5.91988700	-0.21472700

H	-3.86222600	4.41994600	-1.41082800
C	-4.48143200	5.74596300	2.17382800
H	-3.29600900	4.08835300	2.83228100
C	-5.02601800	6.41600900	1.07582600
H	-5.21840900	6.44681500	-1.06159400
H	-4.64294300	6.10051000	3.18403000
O	-5.78872500	7.54512900	1.15183000
C	-6.05817100	8.09801500	2.44172400
H	-6.66985900	8.97978300	2.26258300
H	-6.61013500	7.39127100	3.06776700
H	-5.13396100	8.39068000	2.94793700
N	-0.35308400	-0.57362700	-0.65385500
C	-0.06840000	-1.03264500	-2.02285900
H	1.00017400	-1.19927600	-2.16090900
H	-0.60532400	-1.95574800	-2.25275800
H	-0.39373400	-0.25607300	-2.71191300
C	0.03649600	-1.28153200	0.44172000
O	0.67743400	-2.42470700	0.08326900
O	-0.16556800	-0.95171500	1.59984900
C	1.15703100	-3.27348800	1.15062900
H	1.08086400	-4.28646200	0.75643700
H	0.49635200	-3.17490200	2.01216800
C	2.57749800	-2.93532500	1.55950700
H	2.66241000	-1.90696800	1.90650900
H	2.89613200	-3.61475800	2.34959900
Se	3.83792000	-3.18055500	0.02411000
Se	5.89719300	-2.81406400	1.11666300
C	6.31672300	-4.62313600	1.81951400
H	5.43002800	-5.00273400	2.32645400
H	7.09598600	-4.44364300	2.56446800

C	6.79845500	-5.57997200	0.74687800
H	6.03058700	-5.69914100	-0.02644900
H	7.70585900	-5.18863400	0.27192900
O	7.06165400	-6.83124400	1.39186700
H	7.35767600	-7.45720500	0.72002200
C	-3.15587600	-0.81385000	-0.46525900
H	-2.48837900	-1.65032500	-0.61367800
C	-4.53656200	-1.05959000	-0.43148700
H	-5.18779500	-0.20338500	-0.30950600
C	-6.92441100	-3.76038300	-0.61677300
C	-5.78282300	-4.56888600	-0.71100100
C	-5.91338000	-5.94327100	-0.81558700
C	-7.19778900	-6.50356000	-0.82060200
C	-8.32892500	-5.68627500	-0.72060600
C	-8.21390600	-4.30055900	-0.61585900
C	-5.16700700	-2.29727500	-0.54374100
C	-4.54463300	-3.69312000	-0.68000500
H	-5.03828600	-6.58062800	-0.89193500
H	-7.31628800	-7.57867500	-0.90082600
H	-9.31657300	-6.13509100	-0.72179500
H	-9.09771600	-3.68022500	-0.53132700
C	-3.74959600	-3.84705900	-2.00044700
H	-2.85757600	-3.21998500	-2.00872200
H	-3.43312900	-4.88675000	-2.11240300
H	-4.36633100	-3.58197100	-2.86194900
C	-3.66224200	-4.05756500	0.53885600
H	-2.77307900	-3.42885300	0.59452200
H	-4.21974000	-3.94975700	1.47177900
H	-3.33652900	-5.09683200	0.45240600
N	-6.53478500	-2.42363500	-0.53423000

C	-7.49956000	-1.32393100	-0.41966500
H	-8.39929400	-1.62886400	-0.95418500
H	-7.09959400	-0.46265200	-0.95401500
C	-7.82921500	-0.96942000	1.03157700
H	-6.93886200	-0.64339100	1.57315200
H	-8.55734400	-0.15482600	1.04855200
H	-8.26104100	-1.82416200	1.55665600
H	-1.61116000	3.49144600	-1.29746400
H	-4.27362100	1.45488600	0.47133300
H	-2.31496100	2.50259800	1.50096000

Coordinates for Cy (S_1)

C	7.19738800	-1.31533900	-0.16363900
C	6.70668900	-2.51040900	0.38240100
C	7.58604400	-3.49692500	0.79487600
C	8.96377000	-3.27728000	0.66070600
C	9.44103200	-2.07887200	0.11909800
C	8.56826800	-1.07598200	-0.30181600
C	4.92035100	-1.07703100	-0.18995400
H	7.21914600	-4.42715600	1.21677700
H	9.66488600	-4.04042900	0.98074100
H	10.51029600	-1.92111900	0.02514600
H	8.95207300	-0.15002200	-0.71232300
C	4.66725000	-2.59760400	1.85230700
H	4.99054500	-3.55403600	2.27010400
H	5.06078500	-1.79794700	2.48364800
H	3.57808300	-2.56350300	1.89170600
C	5.19016200	-2.46762900	0.40140500
C	4.61559200	-3.59644200	-0.49012200

H	4.97859700	-3.50845300	-1.51648000
H	4.93258200	-4.56531800	-0.09669700
H	3.52555800	-3.57905700	-0.50741900
N	6.12652000	-0.49332100	-0.50868700
C	6.30724000	0.83653700	-1.09968200
H	7.21583900	0.80128200	-1.70109000
H	5.48537200	1.01615700	-1.79243400
C	6.39805600	1.94846900	-0.05228100
H	7.24204400	1.78603000	0.62157900
H	6.54185700	2.90835100	-0.55434200
H	5.48504500	2.00792300	0.54376900
C	3.70685400	-0.43117500	-0.39368500
H	3.76186300	0.57103100	-0.80018000
C	2.42411600	-0.94974200	-0.12921600
H	2.33990300	-1.96358600	0.23688800
C	1.21325700	-0.26553500	-0.28619700
C	-0.03382800	-0.94069600	-0.07521200
C	1.15354800	1.19877200	-0.64086000
C	-1.26238600	-0.23183700	-0.02459600
C	0.11916300	1.90937300	0.25428700
H	2.12628800	1.67498500	-0.51920800
C	-1.26271800	1.28429300	0.01414200
H	-1.66609100	1.65534900	-0.93690500
C	0.11162400	3.41584200	0.07366000
C	-0.15674000	4.01524500	-1.16849200
C	0.37663300	4.26253400	1.15128200
C	-0.15940000	5.39464200	-1.31978700
H	-0.36761600	3.39780800	-2.03623500
C	0.37680700	5.65445400	1.02013600
H	0.58970500	3.83323900	2.12623000

C	0.10673400	6.22858100	-0.22407300
H	-0.36688200	5.84934200	-2.28295600
H	0.58749000	6.26830100	1.88673900
O	0.07884500	7.57041100	-0.47258000
C	0.33786000	8.46649200	0.61006700
H	0.25942500	9.46706900	0.18998000
H	-0.39978300	8.34622900	1.40852100
H	1.34286700	8.31800900	1.01501500
N	-0.00071100	-2.34272200	0.10738300
C	0.21711500	-3.18283000	-1.08116100
H	0.28052700	-4.22658900	-0.76867500
H	-0.58970100	-3.08536300	-1.81986300
H	1.15645300	-2.91516500	-1.56472900
C	-2.49623400	-0.91614700	-0.03990200
H	-2.46435400	-1.99060800	-0.15502500
C	-3.75846900	-0.30540700	0.02469700
H	-3.78102300	0.77250400	0.12769800
C	-7.27902200	-1.11061800	-0.01299000
C	-6.82495800	-2.43471800	-0.08963200
C	-7.73419600	-3.47811200	-0.13318200
C	-9.10466500	-3.18656400	-0.09549200
C	-9.54464900	-1.86153400	-0.01408600
C	-8.64104900	-0.79953700	0.03005400
C	-4.99562200	-0.94284900	-0.02815800
C	-5.30738000	-2.44353700	-0.11539400
H	-7.39588800	-4.50758300	-0.19500400
H	-9.82854100	-3.99366000	-0.12784200
H	-10.60839900	-1.65041200	0.01826500
H	-8.99573100	0.22157400	0.10068900
C	-4.79963800	-3.06534800	-1.43985200

H	-3.71027200	-3.07215100	-1.48949900
H	-5.14735600	-4.09869500	-1.51209200
H	-5.18075500	-2.51469700	-2.30265100
C	-4.75565600	-3.22443900	1.10191500
H	-3.66518200	-3.23402800	1.11336600
H	-5.10637300	-2.78711600	2.03926300
H	-5.10195200	-4.25973100	1.05652600
N	-6.18258900	-0.24834800	0.00608700
C	-6.32323700	1.20913200	0.08075400
H	-7.25024800	1.46971300	-0.43048200
H	-5.51579400	1.65926300	-0.49667000
C	-6.33512900	1.73654200	1.51698000
H	-5.40319800	1.50009100	2.03472800
H	-6.45303900	2.82287300	1.50403500
H	-7.16480200	1.31022600	2.08515300
H	0.87085400	1.32200300	-1.69450100
H	-1.95215800	1.63957900	0.78678500
H	0.40308800	1.70760900	1.29351200
H	-0.80279700	-2.67330200	0.62776100

Coordinates for Cy-DiSe (T_1)

C	4.99722000	4.39649600	-1.08850400
C	5.34581300	3.04246400	-1.07708200
C	6.67591100	2.66878800	-1.18442900
C	7.65227100	3.66626400	-1.29954000
C	7.28963400	5.01593800	-1.30546300
C	5.95336100	5.40599600	-1.19913800
C	3.00782700	3.28428300	-0.86726700
H	6.96134200	1.62181000	-1.17931600

H	8.69764500	3.38938700	-1.38295900
H	8.05658900	5.77824100	-1.39194900
H	5.68650000	6.45575400	-1.19813000
C	4.14853500	1.35157300	0.34907300
H	4.99968600	0.66842000	0.29935000
H	4.27068700	1.98720900	1.22892500
H	3.24586000	0.75391400	0.47915000
C	4.09093400	2.19935200	-0.94325200
C	3.91951400	1.29799900	-2.18950400
H	3.87080800	1.89669900	-3.10181000
H	4.77434700	0.62243700	-2.26879400
H	3.01556600	0.69136000	-2.12707800
N	3.60335300	4.51505800	-0.97778200
C	2.91993100	5.81386800	-0.94871500
H	3.49543500	6.49374200	-1.57696800
H	1.94908300	5.69999600	-1.42938500
C	2.77446100	6.37807500	0.46511000
H	3.74988600	6.52039400	0.93525900
H	2.27386400	7.34814500	0.41851500
H	2.17871800	5.71617500	1.09720400
C	1.63180000	3.13874200	-0.70006500
H	1.06570600	4.05900500	-0.63494300
C	0.91247300	1.93871900	-0.61572800
H	1.44489300	1.00303500	-0.70888000
C	-0.48409500	1.85141200	-0.44454900
C	-1.14235000	0.59781900	-0.44926300
C	-1.32325800	3.09737600	-0.28289800
C	-2.54754600	0.45235400	-0.34641500
C	-2.60546700	2.83432600	0.52021600
H	-0.74942300	3.88469000	0.20807100

C	-3.38872200	1.69065700	-0.13864900
H	-3.77405500	2.01997100	-1.11260400
C	-3.44353400	4.08576800	0.70504300
C	-3.99501400	4.77978400	-0.38490100
C	-3.69038600	4.59267300	1.98176200
C	-4.75499300	5.92601900	-0.20092600
H	-3.83028900	4.42399600	-1.39734000
C	-4.45370400	5.74563400	2.18823500
H	-3.27883900	4.08060100	2.84696800
C	-4.99136600	6.42070500	1.08993200
H	-5.17697400	6.45681700	-1.04803800
H	-4.61658100	6.09888100	3.19865600
O	-5.74835800	7.55350000	1.16586100
C	-6.01899300	8.10529200	2.45603400
H	-6.62543600	8.99061600	2.27664000
H	-6.57667000	7.40023300	3.07888500
H	-5.09485400	8.39208900	2.96570100
N	-0.34860100	-0.58744300	-0.64713900
C	-0.06702700	-1.04669500	-2.01655500
H	1.00002100	-1.22258300	-2.15516100
H	-0.61105300	-1.96501900	-2.24945400
H	-0.38545700	-0.26596200	-2.70419100
C	0.03976900	-1.30025300	0.44565200
O	0.67652900	-2.44518100	0.08300400
O	-0.15972600	-0.97525500	1.60568200
C	1.15147600	-3.29976200	1.14740200
H	1.07802900	-4.31042700	0.74677000
H	0.48654100	-3.20704000	2.00636400
C	2.56970800	-2.96352500	1.56579400
H	2.65225200	-1.93750600	1.92010500

H	2.88493500	-3.64809500	2.35282000
Se	3.83782700	-3.19762900	0.03499200
Se	5.89135000	-2.83591200	1.13996000
C	6.30680500	-4.64770400	1.83820600
H	5.41758200	-5.02872000	2.33963300
H	7.08259500	-4.47119000	2.58748700
C	6.79316300	-5.60106900	0.76457700
H	6.02894800	-5.71715300	-0.01281800
H	7.70305600	-5.20862400	0.29533800
O	7.05256500	-6.85470100	1.40654000
H	7.35076800	-7.47873500	0.73387400
C	-3.15550800	-0.81457400	-0.46230200
H	-2.49495400	-1.65500800	-0.61651400
C	-4.53710000	-1.04553300	-0.42663800
H	-5.17930900	-0.18411000	-0.29466200
C	-6.95500900	-3.72342900	-0.62814200
C	-5.82404300	-4.53797200	-0.73901000
C	-5.96869200	-5.91084300	-0.86010600
C	-7.25786600	-6.45816900	-0.86417600
C	-8.37910300	-5.63216900	-0.74688700
C	-8.24749200	-4.24759300	-0.62561300
C	-5.18379500	-2.27437400	-0.54822300
C	-4.57533300	-3.67574200	-0.70296700
H	-5.10019100	-6.55542800	-0.94962600
H	-7.38713100	-7.53103200	-0.95724000
H	-9.37165700	-6.06990300	-0.74750900
H	-9.12405600	-3.61881200	-0.52862100
C	-3.78673100	-3.82162500	-2.02649700
H	-2.88843600	-3.20339600	-2.03079200
H	-3.48014600	-4.86261800	-2.15270600

H	-4.40330300	-3.54038700	-2.88311800
C	-3.69487400	-4.06131600	0.50941600
H	-2.79947900	-3.44194700	0.57105900
H	-4.24957600	-3.95860700	1.44470900
H	-3.37855100	-5.10248100	0.41196500
N	-6.55089200	-2.38333500	-0.53181900
C	-7.50543400	-1.27700700	-0.39352700
H	-8.40912300	-1.56426500	-0.93099800
H	-7.10058000	-0.40974500	-0.91377800
C	-7.82567200	-0.94707000	1.06481000
H	-6.93067100	-0.63499300	1.60712100
H	-8.54976700	-0.12963800	1.10132200
H	-8.25835900	-1.80882300	1.57763700
H	-1.58331700	3.47985700	-1.27884500
H	-4.26289900	1.45765600	0.47223500
H	-2.30231300	2.49290900	1.51660000
